# Buprenorphine in rats: potent analgesic or trigger for fatal side effects?

**DOI:** 10.1186/s13028-022-00661-y

**Published:** 2022-12-13

**Authors:** Janin Reifenrath, Miriam Heider, Merle Kempfert, Heidi Harting, Friederike Weidemann, Sarah Strauss, Nina Angrisani

**Affiliations:** 1 Hannover Medical School, Clinic for Orthopaedic Surgery, Carl-Neuberg-Straße 1, 30625 Hannover, Germany; 2 Hannover Medical School, Institute for Laboratory Animal Science and Central Animal Facility, Carl-Neuberg-Straße 1, 30625 Hannover, Germany; 3 Hannover Medical School, Trauma Department, Carl-Neuberg-Straße 1, 30625 Hannover, Germany; 4 Hannover Medical School, Department of Plastic, Aesthetic, Hand and Reconstructive Surgery, Carl-Neuberg-Straße 1, 30625 Hannover, Germany; 5 Present Address: Deutsches Krebsforschungszentrum Stiftung des öffentlichen Rechts, Zentrum für Präklinische Forschung, Im Neuenheimer Feld 280, 69120 Heidelberg, Germany

**Keywords:** Adverse effect, Lewis, Opioid, Pain management, Refinement, Rodent, Sprague Dawley

## Abstract

With ongoing animal welfare efforts, multimodal analgesia is often recommended to implement in study protocols. Buprenorphine with very potent analgesic effect is a standard opioid for the use in this context in rats. In this study, two rat strains (LEW/NHanZtm, n = 6 and Crl:CD(SD), n = 8) underwent orthopaedic surgery and received carprofen, buprenorphine and a local anaesthetic in a multimodal setup. Crl:CD(SD) rats showed severe side effects in the first 24 h after anaesthesia, predominantly manifesting in pica-behaviour and reaching humane endpoints in two of eight animals, while LEW/NHanZtm rats showed only slight depression in the first postoperative days. In the context of improving animal welfare in experimental studies, buprenorphine is highly recommended not to be used in male Crl:CD(SD) rats and should generally be used very carefully and only if required.

**Supplementary Information:**

The online version contains supplementary material available at 10.1186/s13028-022-00661-y.

## Findings

In accordance to refinement procedures, multimodal analgesic therapy is often recommended for surgical procedures. The German Society for Laboratory Animals suggests regimes, which implement nonsteroidal anti-inflammatory drugs for two to five days as well as occasionally necessary opioid use for interventions on extremities or spine with mild to moderate pain and opioid use for interventions with bone implementation (e.g., fracture models); with buprenorphine mentioned in particular [1]. Analgesics does not have only positive effects. Especially opioids can induce side effects, which influence animal welfare negatively. Examples are constipation, respiratory depression, nausea and urinary retention, addiction, tolerance and hyperalgesia [2]. In rats, nausea seems to potentially manifest in the so-called pica-behaviour, which is expressed by the uptake of inedible material like wooden embedding or cellulose in larger amounts. Especially Sprague Dawley (SD) rats are mentioned in reports concerning pica-behaviour [3, 4], however severe side effects with reaching of humane endpoints is not mentioned in these studies.

The here presented data result from two pilot studies for stress evaluation, which used multimodal analgesia with carprofen, buprenorphine and lidocaine hydrochloride in male Lewis rats (strain LEW/NHanZtm) (study 1) and SD rats (strain Crl:CD(SD)) (study 2) (Table 1).

**Table 1:** Summarized study design and detailed information on implemented animals

Study	Strain	Sex	Age	Body weight	n	Surgical procedure	Follow-up
1	LEW/NHanZtm^a^	Male	> 16 week	410.8 ± 8.3 g	6	intervertebral disc puncture	6 week
2	Crl:CD (SD)^b^	Male	> 16 week	547.6 ± 43.7 g	8	intramedullary pin insertion	3 week

^a^Central Animal Facility, Hannover Medical School, Germany

^b^Charles River Laboratories, Sulzfeld, Germany

The pre-emptive multimodal analgesic protocol before surgery and in the first 12 h after surgery was identical in both studies and implemented carprofen, buprenorphine and local anaesthetic with a second buprenorphine injection 4–8 h after the pre-emptive administration (Table 2).

**Table 2:** Multimodal analgesic regime for both studies summarizing the pharmaceutics used and respective application time points

	Day of surgery			1st day post operative			2nd–5th day post operative
	Pre-emptive	4–8 h post surgery	12 h post surgery	Morning	Middle of day	At night	Morning
LEW/NHanZtm (study 1)	Car, Bup, LA	Bup	–	Car	–	–	Car
Crl:CD(SD) (study 2)	Car, Bup, LA	Bup	Bup	Car, Bup	Bup	Bup	Car

The multimodal analgesic regime included the non-steroidal antiphlogistic drug Carprofen (Car, 5 mg/kg s.c.), the opioid Buprenorphine (Bup, 0.05 mg/kg s.c.) and a local anesthetic (LA, lidocaine hydrochloride 2%)

Detailed study protocols are provided as Additional file 1.

After surgery, the animals were under continuous observation until being fully awaken. After appearance of unusual behaviour as reduced or hyperactivity or pica, observation time was extended up to approx. 75 min after the end of surgery.

In study 1, two out of six LEW/NHanZtm rats showed long periods of more than 20 min until complete awakening. One rat needed approximately 40 min, another approximately 60 min. Up to three rats had reduced activity during the first postoperative days. Three out of six animals showed this behaviour on day 1 and/or day 2 after surgery. The condition continued until day 3 for two of these rats. One animal had a slightly shaggy look on day 1, one animal on day 2. The postoperative course was in other ways unapparent. Other animals of study 1 showed no abnormalities in general condition or behaviour.

In study 2, which included eight Crl:CD(SD) rats, three rats showed prolonged periods of 30 up to 45 min until awakening of anaesthesia after end of surgery, while the remaining five animals were fully awake at the latest 20 min after isoflurane inhalation has stopped. Rat nos. 2 and 5 showed spontaneous uptake of cellulose and/or bedding material while rat no. 7 showed pronounced chewing. Hyperactivity, which was very pronounced in one rat, was observed in all animals.

Rat no. 5 showed a slight dehiscence of the sutures, which were then revised. This animal showed a reduced spontaneous activity the next morning. Following these, no further clinical signs were observed.

**Fig. 1:**
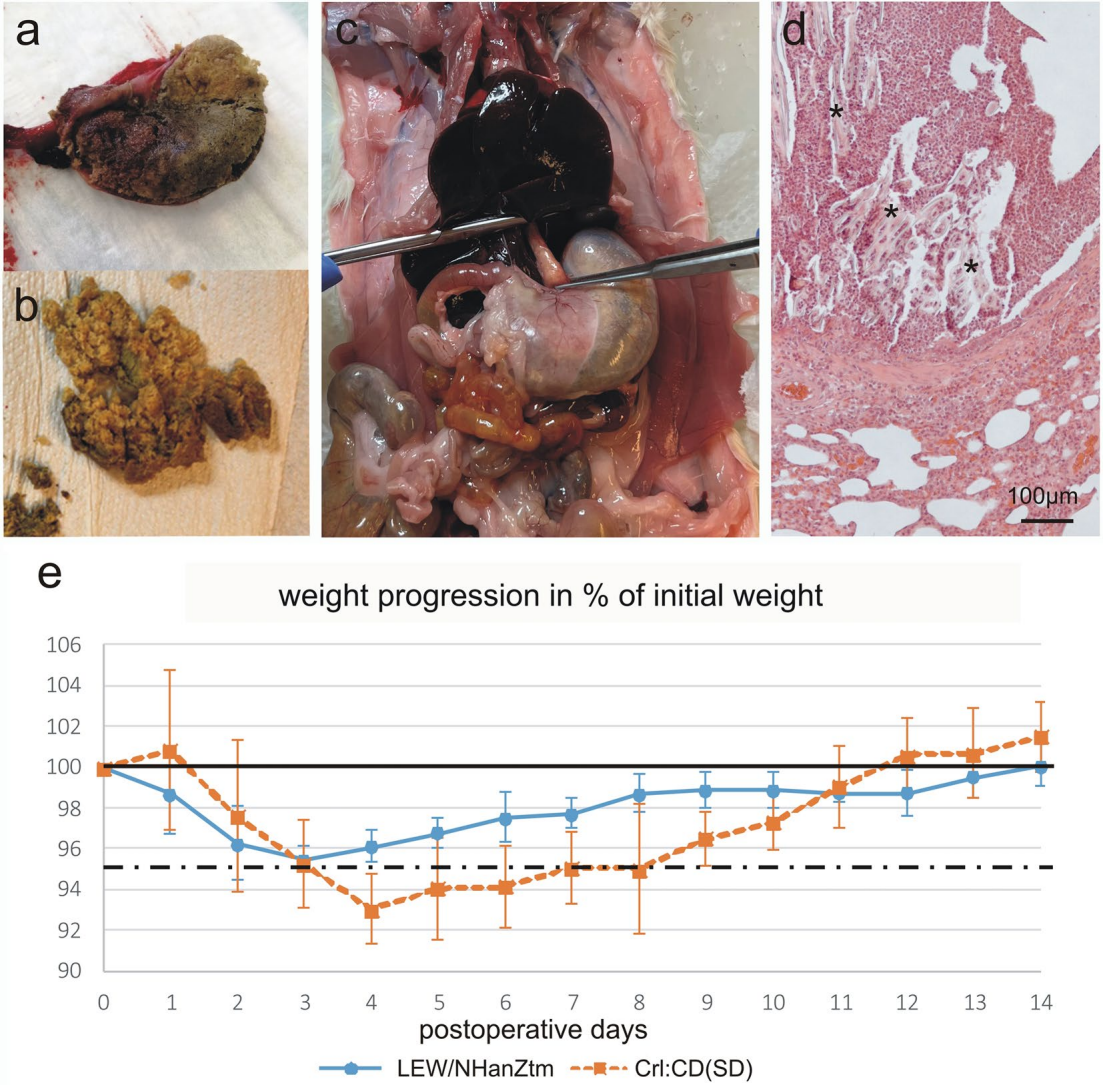
**a** The stomach has been opened along the greater curvature and a large solid content mainly consisting of embedding material is seen. Crl:CD(SD) rat no. 6, which showed also automutilation; **b** A large amount of stomach content mainly consisting of embedding material. Crl:CD(SD) rat no. 8, which also developed dyspnoe; **c** The abdomen organs are exposed after opening the abdominal wall. A severely distended stomach is seen. Crl:CD(SD) rat no. 8; **d** Photomicrograph of lung tissue showing an aspirations bronchopneumonia. Foreign material is indicated by *. Hematoxylin and eosin stain, Crl:CD(SD) rat no. 8; **e** Graph showing the development in body weight in % of starting weight for the two groups of rats

Rat no. 6, which was in the same cage as rat no. 5, showed automutilation distal to the surgical wound at the time of the scheduled second injection. A 2–3 cm area of skin and musculature at the craniolateral aspect of the tibia was chewed at and partly eaten away. The rat was humanely euthanized. Necropsy revealed a severely distended stomach with a content mainly composed of embedding material (Fig. 1a).

Rat no. 8 showed dyspnea the morning of the first postoperative day expressed as abdominal breathing and neck stretching with each breath. Dyspnea got worse in the next hour and the rat reached humane endpoint and was humanely euthanized. Necropsy revealed severely dilation of the distal portion of the oesophagus and the stomach due to accumulation of embedding material (Fig. 1b and c).

Development in body weight (BW) differed between study groups. In Crl:CD(SD) rats with repeated buprenorphine injections, an increase in BW between 15 and 25 g on the first postoperative day was observed in three out of six animals, followed by a significant decrease in BW until day four. In LEW:NHanZtm rats with a single postoperative buprenorphine injection, also three out of six animals showed an increase in BW on the first postoperative day, but in a significantly lower range (1–3 g). Taken all animals of this group into account, BW on the first postoperative day decreased distinctly, followed by a less pronounced further decrease in BW until day 3 (Fig. 1e).

Severe side effects of subcutaneous buprenorphine exposure in the first hours after surgery were observed in Crl:CD(SD) rats, whereas LEW:NHanZtm rats showed better compatibility; especially differences in pica behaviour was noticeable, which was observed in three Crl:CD(SD) rats as soon as they woke up after anaesthesia. The pica behaviour was reflected in the necropsy findings and considered as a major cause of reaching humane endpoint. An additional particular serious side effect was automutilation, which developed in one Crl:CD(SD) rat.

In humans, self-injurious behavior has been reported in individuals with neurodevelopmental disabilities [5]. There is growing evidence that a disturbance of the opiate-mediated pain and pleasure system is involved with insensitivity to pain and general sensory depression being one possible reason [5]. In animal studies, most reports of automutilation are associated with peripheral nerve injuries (e.g. [6]). While the initial trigger for the automutilation in one of the Crl:CD(SD) rats is not evident, possible reasons might be irritation caused by the suture or paraesthesia due to lesions of nerves, although the latter seems unlikely as consequence of the intervention performed. However, the pain during automutilation should have prevented further progression. Buprenorphine might have contributed to reduced sensory processing or even pain absence, which may have led to the severe manifestation of automutilation.

In contrast, none of the LEW:NHanZtm rats developed automutilation and pica-behaviour in the comparable postoperative period despite similar medication. That allows us to conclude that strain specific differences in the expression of buprenorphine-induced side effects exists. LEW:NHanZtm rats were slightly reduced in general condition or activity whereas Crl:CD(SD) rats showed pica-behaviour and a tendency to hyperactivity. Strain specific differences regarding pica-behaviour were also described by Thompson et al. [4]. They administered 0.05 mg/kg buprenorphine subcutaneously in SD and Ewans-rats. Ewans-rats did not show any signs of pica-behaviour or wood shavings in the stomach two hours after buprenorphine administration, while wood shavings were found in 80% of SD rats. However, the study was limited to a period of two hours after injection and the authors did not comment, if pica-behaviour severely affected animal welfare or was just an observation without any further impact. Thompson et al. recommended to use the minimal effective subcutaneously administered dose and pose the question, if a reduction of 0.05 mg/kg might be possible. Severe affection of animal welfare and subsequent euthanasia is described only once by Boosgraf et al. for Wistar rats after surgery [7]. Berke et al. described an arthritis model in SD rats (NTac:SD), where buprenorphine was administered subcutaneously (0.05 mg/kg) twice a day compared to carprofen (5 mg/kg) once a day. They did not report any adverse effects. Paw withdrawal latency times in an electronic von Frey test and rat grimace scale evaluation as signs for pain after adjuvant induced monoarthritis did not differ between treatment groups [8]. However, it is questionable, if buprenorphine injection twice daily is sufficient for an efficient analgesic treatment in rats, as Goldkuhl et al. [9] showed that buprenorphine plasma concentration after 0.05 mg/kg subcutaneous injection was close to zero after 6 h in Wistar and SD rats. Allen et al. [10] examined self-injury after buprenorphine administration in doses between 0.075 and 0.3 mg/kg administered once subcutaneously in healthy adult male SD rats. In their study, animals showed occasional to frequent self-biting with no evidence of skin trauma up to mild to moderate skin trauma in all treatment groups. Food uptake was increased during the light period compared to healthy controls with dextrose injection. Pica-behaviour could not be evaluated as rats were housed in wire-bottom cages during the study.

In our studies, an increase in BW was observed on the first postoperative day in Crl:CD(SD) rats, which was not observed in LEW:NHanZtm rats probable induced by the excessive uptake of food and other materials in the first postoperative hours. Similar increase in BW was also reported by Clark et al. [3]. Therefore, animals which exhibit an increase in BW on the postoperative day should be monitored attentively. In total, Crl:CD(SD) rats lost more BW over 4 days than LEW:NHan rats, which started to gain BW again on day three post surgery. Berke et al. observed a reduced BW after buprenorphine administration and could not show an advantage in buprenorphine administration on rat welfare [8].

In conclusion, buprenorphine is a challenging analgesic and serious side effects may develop. Following our observations and the referenced literature, contrary to other strains, especially SD rats seem to pose a risk to develop pica-behaviour. Although this finding is generally known, little to none could be found about the impact on reaching of humane endpoints, which was observed in this study. Due to this fact, the authors highly recommend to avoid buprenorphine use in male SD rats. Further studies are needed to clarify the impact of animal strain and sex or buprenorphine doses on the severity of side effects while maintaining adequate analgesic levels.

## Supplementary Information

**Additional file 1. **Study details.

## Data Availability

The datasets used and/or analyzed during the current study are available from the corresponding author on reasonable request.
