# Supplementary material for: Buprenorphine in rats: potent analgesic or trigger for fatal side effects?
Source: Acta Vet Scand. 2022 Dec 13;64:37. doi: 10.1186/s13028-022-00661-y (PMC9749369; doi:10.1186/s13028-022-00661-y)
Supplement: Supplementary file 1 — Additional file 1. Study details. [file 13028_2022_661_MOESM1_ESM.docx]

**Additional file 1. Study details**

All rats were monitored according to the FELASA guidelines [1], housed in pairs in open cages (type IVs, Uno Roestvaststaal BV, The Netherlands;), temperature between 20 °C and 24 °C, relative humidity of 45–65% and 14/10h day–night cycle with free access to standard diet (1324 TPF, Altromin, Lage/Westphalia, Germany) and water. The adaption time prior to surgery was at least 14 days.

Rats received carprofen (5 mg/kg s.c. Rimadyl®, Zoetis, Berlin, Germany) at least 30 min and buprenorphine (0.05 mg/kg s.c., Bupresol® vet, cp-Pharma, Burgdorf, Germany) approximately 15-20 min before surgery. Anaesthesia was performed by isoflurane (2–5 vol %; Isofluran CP, cp–pharma, Burgdorf, Germany) in oxygen. Animals were placed on a heating mat. Bepanthen eye ointment was applied. Warmed infusion solution (5-10 mL/kg) was administered subcutaneously.

Local anaesthetic (Lidocainhydrochlorid 2%, bela-pharm, Vechta, Germany) was infiltrated subcutaneously at the tail base (10 mg/kg, study 1) or intraarticular (5 mg/kg) in the knee joint (study 2).

***Surgical procedure***

*Study 1:* The rats were placed in prone position. An elastic band was placed at the tail base to avoid bleeding. The dorsal tail was incised approximately 3 cm above the vertebral segments Co4-5 to Co6-7. Muscles and tendons were carefully displaced and the dorsal parts of two neighbouring intervertebral discs were exposed and incised with an 18G cannula. One disc was filled with a biomaterial whereas the other was left empty. Wound closure was performed in a single layer with an interrupted suture pattern (Vicryl 4/0, Ethicon, Johnson & Johnson Medical N.V., Belgium).”

*Study 2*: The rats were placed in supine position. An incision was made medially at the left knee, the patella luxated laterally. A 17G cannula was inserted in the intercondylar notch, protruded intramedullary and replaced by a pin (Ø 0.5mm, 25 mm length). The knee joint was flushed with saline solution and closed with layered suture (Monosyn 6/0, Ethicon, Johnson & Johnson Medical N.V., Belgium).

Enrofloxacin (5 mg/kg s.c., Bayer, Leverkusen, Germany) was administered in both groups prior to surgery and in study 1 additionally for four further days.

***Evaluation of animals***

General condition and behaviour were scored daily during the first 14 postoperative days (table below). Additionally, body weight, surgical wound and in study 2 lameness were evaluated.

Score scheme for the evaluation of general condition and behaviour

| **Score value** | | | | |
| --- | --- | --- | --- | --- |
|  | **1** | **2** | **3** | **4** |
| **General condition** | Coat smooth, shiny, orifices clean, defecation and urine secretion normal, eyes bright | Focally shaggy coat, defecation and urine secretion normal | Coat matted, shaggy, neglected grooming of orifices, defecation and urine secretion slightly affected | Dirty coat, sticky or moist orifices, defecation and urine secretion impaired, atypical body posture, eyes sticky and sunken |
| **Behaviour** | Awake, attentive, curious, physiological body posture and gait | Calm, attentive, allotriophagy (pica-behaviour), reduced movement, reduced cleaning behaviour, species specific body posture | Very calm, inattentive, weak, sternoabdominal posture, reduced motion | Apathetic, lateral posture, pronounced hyperkinetic or stereotypes of behaviour, coordination disorders, automutilation |

**References**

1 FELASA working group on revision of guidelines for health monitoring of rodents and rabbits, Mahler Convenor M, Berard M, Feinstein R, Gallagher A, Illgen-Wilcke B, et al. FELASA recommendations for the health monitoring of mouse, rat, hamster, guinea pig and rabbit colonies in breeding and experimental units. Lab Anim. 2014;48:178-92.
